# Small‐molecule‐driven direct reprogramming of Müller cells into bipolar‐like cells

**DOI:** 10.1111/cpr.13184

**Published:** 2022-01-18

**Authors:** Pan Yang, Qilong Cao, Yani Liu, KeWei Wang, Wei Zhu

**Affiliations:** ^1^ Department of Pharmacology School of Pharmacy Qingdao University Qingdao China; ^2^ Qingdao Haier Biotech Co. Ltd Qingdao China; ^3^ Institute of Innovative Drugs Qingdao University Qingdao China; ^4^ Shenzhen Ruipuxun Academy for Stem Cell & Regenerative Medicine Shen Zhen China; ^5^ Beijing Advanced Innovation Center for Big Data‐Based Precision Medicine Beihang University & Capital Medical University Beijing China

**Keywords:** bipolar cells, direct lineage reprogramming, Müller cells, small molecules

## CONFLICT OF INTEREST

All authors declare that they have no conflict of interest.

## AUTHOR CONTRIBUTIONS

PY, KW, and WZ designed the study. PY, QC, and YL performed the experiments and analyzed the data. PY and YL wrote the manuscript. KW and WZ revised the manuscript.

To the editor,

Stem cell‐based therapy is currently an attractive approach for tissue regeneration and function restoration in both the central nervous system (CNS) and retina.^1^, ^2^, ^3^, ^4^ While this is a reliable and encouraging technique, there are many issues for its clinical application, for example, the non‐autologous cell origin of some stem cells and the potentials for contamination during cell culture.^5^, ^6^ Even the autologous induced pluripotent stem cells, long‐term cell culture *in vitro* may lead to some epigenetic alterations, which can cause some immune reactions after transplantation.^7^ Therefore, an alternative approach, *in vivo* glial reprogramming, has become a new option for replacing the neuronal loss.^8^

Astrocyte, a specialized glial type, is typically used for *in vivo* reprogramming in CNS. When an injury happens in CNS, most astrocytes in the injury site de‐differentiate and generate more astrocytes to form a glial scar.^9^ Intriguingly, a few astrocytes can spontaneously differentiate into neurons.^10^ Through investigating pathways involved, some neurogenic transcription factors (TFs) have been identified and tested for converting glial and neuronal cell fate *in vivo*.^11^ In addition, small‐molecule‐driven reprogramming targeting these TFs has also been developed and applied in CNS.^12^ Müller cells in retina and astrocytes in CNS have many features in common, such as their origins, functions, and stem cell characteristics.^13^, ^14^ In the retinal injury site of Zebrafish, Müller cells de‐differentiate rapidly, proliferate, generate new neural stem cells, migrate to the damaged site, and eventually differentiate into neurons.^15^, ^16^ Therefore, it might be reasonable to predict Müller cells with the same potential for *in vivo* reprogramming and replacing loss of neurons associated with retinal degeneration disease.^17^, ^18^ Researchers from two groups have successfully reprogrammed Müller cells through direct‐lineage reprogramming *in vivo* and generated the functional bipolar cells and retinal ganglion cells (RGC).^19^, ^20^ However, whether small molecule compounds can directly reprogram Müller cells into retinal neurons remains elusive.

As reported by Ma, Y. et al., the use of a neuronal introduction medium consisting DFICBY (dbcAMP, Forskolin, ISX9, CHIR99021, I‐BET151, and Y‐27632) successfully reprogrammed astrocytes into neurons.^12^ Here, we validated this small‐molecule‐driven direct reprogramming in our lab by reprogramming astrocytes from cerebra of C57BL/6J (Figure S1A), which were detected with a robust expression of astrocytes marker—glial fibrillary acidic protein (GFAP) (Figure S1C) and the negligible expressions of neuronal markers—beta III Tubulin (TUJ1) and microtubule association protein‐2 (MAP2) (Figure S1D). Following reprogramming for 16 days, cells not only showed a neuron‐like morphology with a soma cell body and synapses (Figure S1B) but also significantly expressed TUJ1 and MAP2 (Figure S1E). Additionally, the electrophysiological features of chemically induced neurons (CiNs), were investigated using the whole‐cell patch‐clamp technique, showing action potentials (APs) and inward currents (Figure S1E,F).^21^ Overall, small‐molecule‐driven direct reprogramming could generate neuron‐like cells with neuronal morphology and features from astrocytes.

**FIGURE 1:**
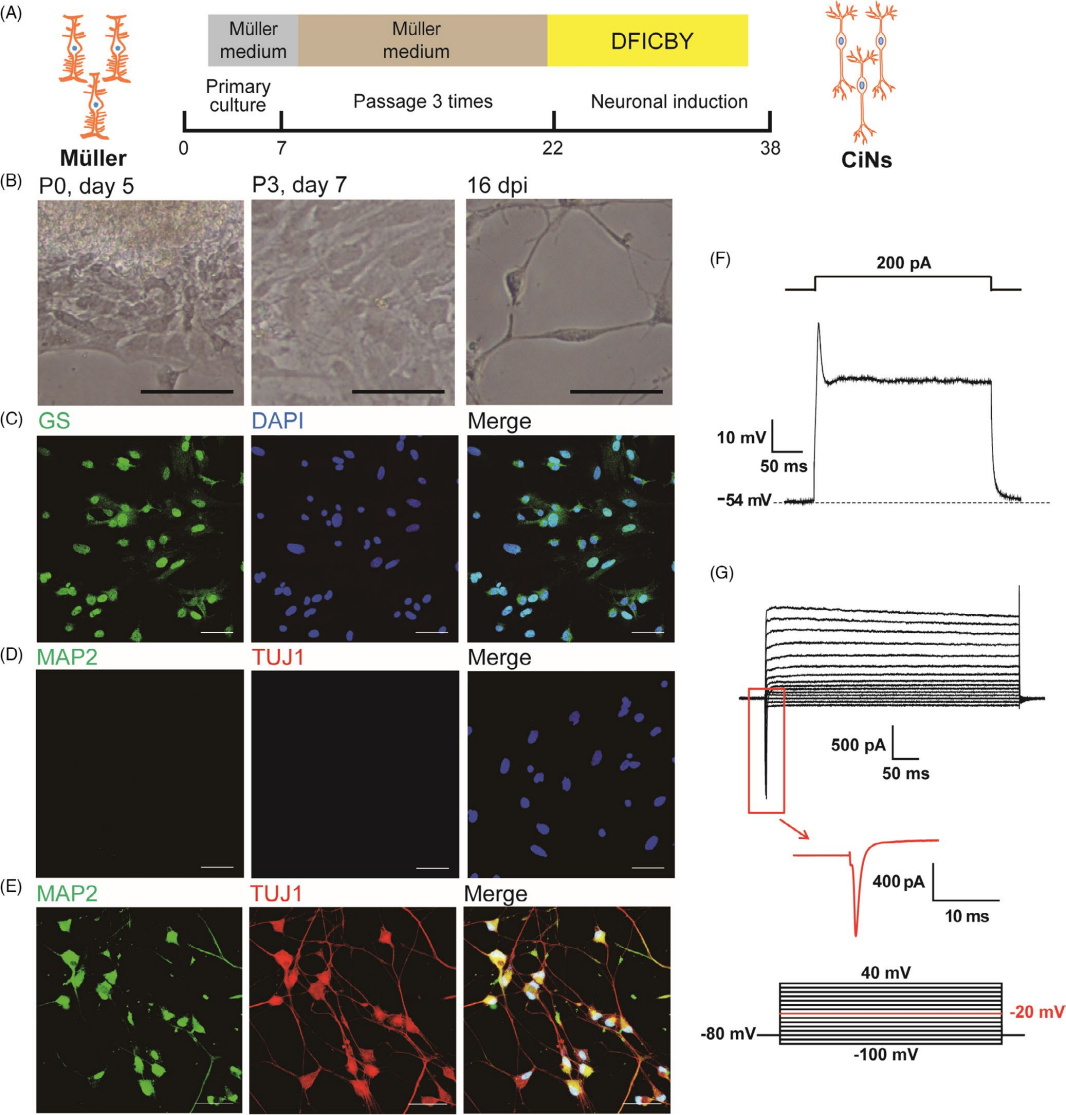
Chemical cocktail efficiently converts Müller cells into bipolar‐like cells *in vitro*. A, Scheme of chemically induced Müller cells to neurons. Primary cultured Müller cells were passaged for three times and then cultured with neuronal induction medium consisting of small molecules DFICBY (100 μM dbcAMP, 10 μM Forskolin, 40 μM ISX9, 20 μM CHIR99021, 2 μM I‐BET151, and 10 μM Y‐27632). B, Light microscopy images of cells at different periods. Left panel: primary culture of Müller cells at day 5, migrating from the retinal tissue. Middle panel: Müller cells at 100% confluency at passage 3. Right panel: chemically induced neurons (CiNs) after 16 days post‐induction (dpi). Scale Bars = 100 μm. C, Immunofluorescence (IF) analyses of GS (red) in Müller cells. D, IF analyses of neuronal markers MAP2 and TUJ1 in Müller cells. E, IF analyses of MAP2 (green) and TUJ1 (red) in Müller cell‐derived CiNs. Nuclei are labeled with 4′,6‐diamidino‐2‐phenylindole (DAPI, blue). Scale Bars = 60 μm in panels C, D, and E. F, Representative induced APs by injecting 200 pA currents. G, Representative current traces elicited by a family potential. A large inward Na^+^ current is observed at −20 mV (red)

Our goal is to generate retinal neurons from Müller cells (Figure 1A). In subsequence, we isolated retinal Müller cells with typical morphology (Figure 1B), the robust expression of Müller cells marker—glutamine synthetase (GS) (Figure 1C) and the negligible expressions of TUJ1 and MAP2 (Figure 1D). Like the above observations, neuronal induction could also induce Müller cells to differentiate into neuron‐like cells with typical bipolar neuronal morphology (Figure 1B) and the robust expressions of both TUJ1 and MAP2 (Figure 1E). Only with the positive staining of TUJ1 and MAP2 expressed by both cerebrum and retina neurons, we still cannot fully understand the type of reprogrammed cell.^22^, ^23^ We consequently investigated electrophysiological features, including APs, resting membrane potential (RMP), and amplitude of APs in Müller cell‐derived CiNs. Although Müller cell‐derived CiNs could not generate typical APs, a single spike could be observed during depolarizing pulses (Figure 1F). To further determine the cell type of these CiNs, we compared the electrophysiological features of amacrines, bipolar cells and RGC (Table 1).^24^, ^25^, ^26^ Given the RMP (−46.5 ± 4.5 mV) and the mean amplitude of APs (48.1 ± 3.5 mV) in our observation, we concluded that Müller cell‐derived CiNs might be more like bipolar cells reported by Puthussery, T. with single AP, RMP of −64 to −55 mV, and amplitude of APs from 30 to 50 mV (Table 1).^25^ In addition, inward current (up to 3 nA; Figure 1G), mainly Na^+^ current, confirmed that they might be DB4 bipolar‐like cells.^25^ Other inner retinal cells show different electrophysiological features (Table 1). Amacrines generate APs spontaneously with a frequency of ~2 to 8 Hz or with burst firing.^24^ RGCs generate serial APs similar to neurons in the cerebrum.^26^ The RMP ranged from −70 mV to −60 mV, and the amplitude of APs was about 100 mV in RGC. Besides, we also reviewed the electrophysiological characteristic of Müller cells. The Müller cells possess large K^+^ channels mediating both inward and outward currents and with small Na^+^ currents. Only under pathological conditions, Müller cells could generate action‐potential‐like activity because Na^+^‐to‐K^+^ conductance ratio becomes very high.^27^, ^28^

**TABLE 1:** Electrophysiological features of cells

	CiNs^†^	Bipolar cells^25^	Amacrines cells^24^	Ganglion cells^26^	Müller cells^27^, ^28^
APs	Single	Single	Spontaneously	Serial	None
RMP (mV)	−57 to −41	−64 to −55	−65 to −30	−70 to −60	−84 to −78
Amplitude of APs (mV)	39 to 60	30 to 50	5 to 90	Around 100	None

† All data from this study.

Small‐molecule‐driven direct reprogramming meets the needs for clinical translation due to its outstanding advantages. The first is its high efficiency in generating neurons from Müller cells, 88.5% of MAP2+/TUJ1+ cells and 55.6% (5 out of 9) cells with the electrophysiological features as bipolar (Figure 1). In contrast, overexpressing Ascl1 generates approximately 30% TUJ1+/orthodenticle homeobox 2+ cells.^29^ Additionally, 12–16 days of reprogramming *in vitro* is sufficient to generate mature bipolar cells (Figure 1), which provides a potential for clinical translation. The direct conversion from glial cells to neurons lowers the risk of teratoma formation compared to the use of induced pluripotent stem cells. Moreover, its low cost, simple synthesis and save and low risk of immune rejection are also attractive for the clinical application. We believe that small‐molecule‐driven direct reprogramming provides a potential for generating bipolar cells *in vivo*. Its use should ultimately facilitate the therapeutic strategy for retina degeneration diseases.

## Supporting information

Figure S1

Supplementary Material

## Data Availability

The authors declare that all the data supporting the findings of this study are available within the article and its Supplementary Information and from the corresponding authors on reasonable request.
