# Supplementary figures and images for: Small‐molecule‐driven direct reprogramming of Müller cells into bipolar‐like cells
Source: Cell Prolif. 2022 Jan 18;55(2):e13184. doi: 10.1111/cpr.13184 (PMC8828256; doi:10.1111/cpr.13184)

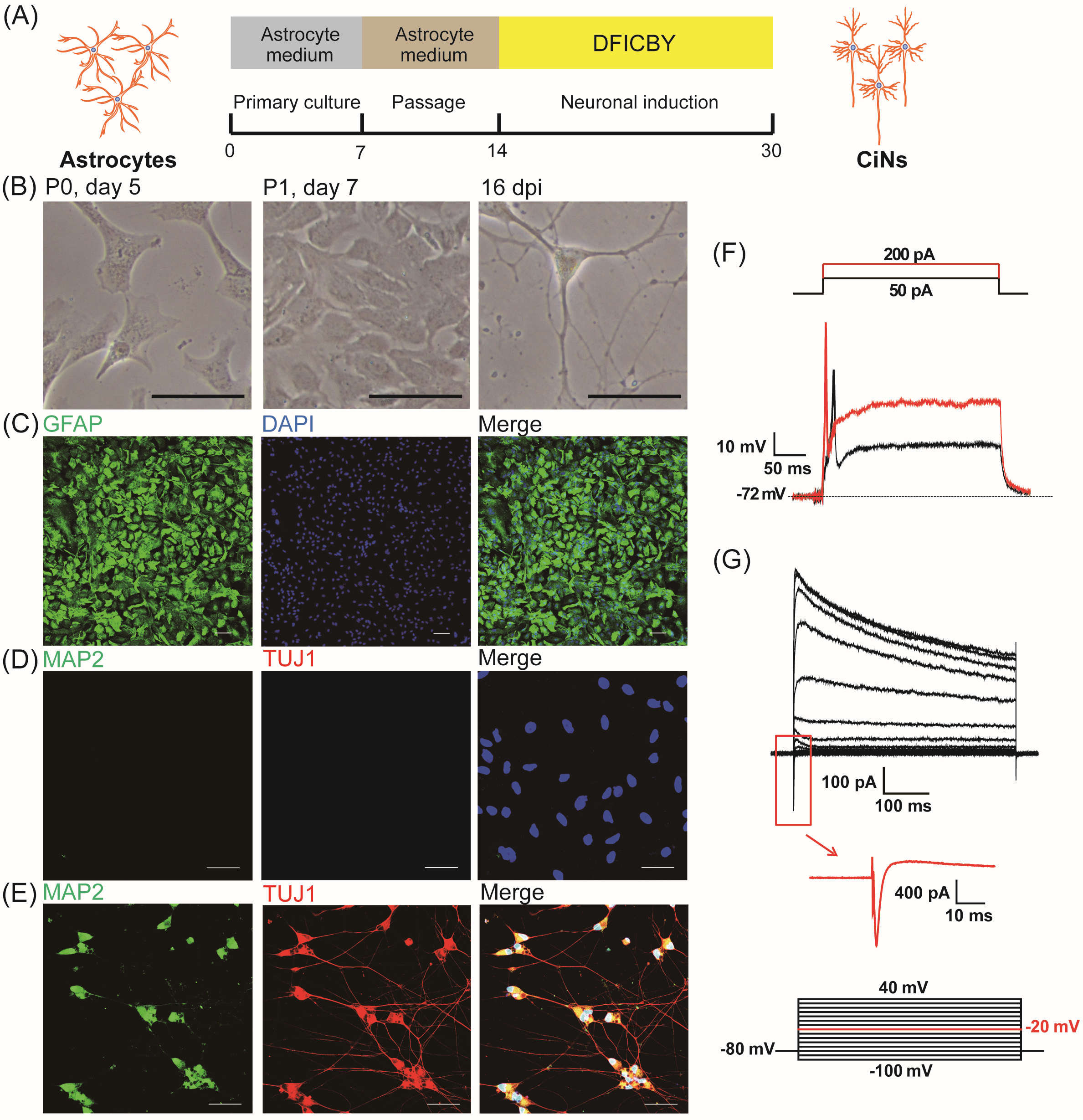

Supplement: Supplementary file 1 — Figure S1 [file CPR-55-e13184-s002.png]
