# Supplementary material for: Small‐molecule‐driven direct reprogramming of Müller cells into bipolar‐like cells
Source: Cell Prolif. 2022 Jan 18;55(2):e13184. doi: 10.1111/cpr.13184 (PMC8828256; doi:10.1111/cpr.13184)
Supplement: Supplementary file 2 — Supplementary Material [file CPR-55-e13184-s001.docx]

**Supplementary Information**

**Materials and Methods**

***Animal***

Pregnant C57BL/6J were purchased from Beijing Vital River Laboratory Animal Technology Co, Ltd (Beijing, China) and housed under standard conditions with a 12 h/12 h day/night cycle (lights on at 7 am and off at 7 pm), at 23 ± 2 °C temperature and 50 ± 5% humidity. All animal protocols were approved by the Institutional Animal Care and Use Committee at Qingdao University and efforts were made to minimize animal suffering and the number of animals used.

***Isolation and culture of astrocytes***

Postnatal C57BL/6J mice (Day 0) were sterilized in 75% alcohol (Sinopharm Chemical Reagent Co. Ltd., Shanghai, China) and decapitated. Cerebra was isolated carefully in the biosafety hood and rinsed with pre-cooled 1×PBS (Gibco, Grand Island, New York). After removing the meningeal, the cerebra was cut into pieces and digested using 0.05% Trypsin (1:5 dilution ratio with PBS; Thermo Scientific, Pittsburgh, PA) for 6 minutes at 37 °C. The reaction was terminated using the astrocyte culture medium comprising of Dulbecco's Modified Eagle Medium/F12 (DMEM/F12) medium (Gibco), 10% fetal bovine serum (FBS; Gibco), and 1% penicillin-streptomycin (Gibco). Medium with gently pipetted tissues was passed through a strainer (100 μm; Millipore, Danvers, MA, USA). After centrifugation at 800 rpm for 5 minutes, the pellet was resuspended in the culture medium, seeded on a poly-D-lysine (Sigma, MO, USA)-coated culture flask (Corning, Corning, USA), and cultured in an incubator at 37 °C, 5% CO_2_. The medium was changed every three days. When the confluency was about 100%, the flask was placed on a shaker (260 rpm, 37 °C) for 18 hours to remove the unattached cells.^1^ The attached cells, known as astrocytes, were collected for characterization and reprogramming.

***Isolation and culture of Müller cells***

Eyes of postnatal C57BL/6J mice (Day 4-5) were enucleated, sterilized in Betadine (Sinopharm Chemical Reagent Co. Ltd.) for 2 minutes, and rinsed in 1×PBS (Gibco). After removing the anterior segment, the retina was isolated from the eyecup and digested with 0.25% Trypsin (Thermo Scientific) for 10 minutes at 37 °C. The reaction was terminated using the Müller cells culture medium consisting RPMI 1640 medium (Gibco), 20% FBS (Gibco), and 1% penicillin-streptomycin (Gibco). The digested tissue was subsequently centrifuged at 1000 rpm for 5 minutes, seeded on a culture flask (Corning) in the Müller cells culture medium, and maintained in an incubator at 37 °C, 5% CO_2_. The medium was changed every three days. When the confluency was about 100%, cells were passaged using 0.25% Trypsin (Thermo Scientific) at a ratio of 1:2. Through passaging, Müller cells was gradually purified after 2-3 passages and used for reprogramming.^2^

***Small-molecule-driven direct reprogramming***

Astrocytes or Müller cells were passaged with 0.25% Trypsin (Thermo Scientific) for 3-6 minutes at 37 °C, seeded on the matrigel (1:100; Corning)-coated 6-well plate (100,000 cells/well; Corning) and maintained in the culture medium. At a confluence of 100%, cells were cultured with neuronal induction medium consisting of neurobasal (Gibco), 0.5% N2 (Gibco), 1% B-27 (Gibco), 1% GlutaMAXTM-I (Gibco), 1% penicillin-streptomycin (Gibco), basic fibroblast growth factor (bFGF; 100 ng/ml; Origene, Rockville, USA), and small molecules as follows: dbcAMP (100 μM; Santa Cruz Biotechnology, Dallas, USA), Forskolin (10 μM; Enzo Life Science, Farmingdale, USA), ISX9 (40 μM; Tocris, Minneapolis, USA), CHIR99021(20 μM; Tocris), I-BET151 (2 μM; Tocris), and Y-27632 (10 μM; Tocris).^1^ After neuronal induction for two weeks, cells were used for immunofluorescence and electrophysiological analyses.

***Immunofluorescence (IF)***

Cells were rinsed with 1×PBS (Gibco) and fixed in 4% (wt/vol) paraformaldehyde (PFA; Sinopharm Chemical Reagent Co. Ltd.) for 20 minutes at room temperature. After treating with 0.3% Triton X-100 (Sigma) for 10 minutes, samples were incubated with the block solution comprising of 1×PBS (Gibco), 1% bovine serum albumin (BSA; Sigma), 0.3% Triton X-100 (Sigma) for 1 hour at room temperature. Samples were then reacted with the diluted primary antibodies at 4 °C overnight followed by the corresponding secondary antibodies for 1 hour. The nuclei were stained with 4’,6-diamino-2-phenylindole (DAPI; Invitrogen Corporation, Carlsbad, CA; 1:1000). Samples were mounted using ProLong Gold Antifade reagent and imaged by confocal microscopy (Nikon; Nikon Corp, Tokyo, Japan).^3^

The primary antibodies included mouse monoclonal anti-glial fibrillary acidic protein (GFAP, Abcam, ab10062, 1:100), rabbit polyclonal anti-beta III Tubulin (TUJ1, Abcam, ab18207, 1:200), chicken polyclonal anti-microtubule association protein-2 (MAP2, Abcam, ab5392, 1:500), mouse monoclonal anti-glutamine synthetase (GS, Novus, NBP2-43636, 1:100). The secondary antibodies were Alexa Fluor® 488 goat anti-mouse immunoglobulin G (IgG; Invitrogen), Alexa Fluor® 488 goat anti-chicken IgG (Invitrogen), and Alexa Fluor® 568 goat anti-rabbit IgG (Invitrogen). The dilution ratio of the secondary antibodies was 1:200.

***Electrophysiology***

At 16 days post-induction, chemically induced neurons were recorded using a whole-cell patch-clamp configuration. The recording electrodes had a resistance of 2 to 6 MΩ when filled with the pipette solution containing (mM): K-gluconate 100, KCl 50, EGTA 10, MgCl_2_ 5, and HEPES 2 at pH 7.3 adjusted with KOH. The extracellular solution consisted (mM) NaCl 140, KCl 3, CaCl_2_ 2, MgCl_2_ 2, HEPES 10, and glucose 10 at pH 7.4 adjusted with NaOH. To record action potentials and inward currents, the extracellular solution was supplemented with ionotropic glutamate NMDA and non-NMDA receptor inhibitors DL-2-amino-5-phosphonovaleric acid and 6,7-dinitroquinoxaline-2,3-dione (each at 20 μM), and GABA receptor inhibitors bicuculline and baclofen (each at 10 μM).^4^ All experiments were carried out in an air-conditioned room with a controlled temperature of 22 ± 1 °C.

**Statistical analysis**

All data were expressed as the means ± SEM.

**References**

1. Ma Y, Xie H, Du X, et al. In vivo chemical reprogramming of astrocytes into neurons. *Cell Discov.* 2021;7(1):12.

2. Liu X, Tang L, Liu Y. Mouse Müller Cell Isolation and Culture. *Bio Protoc.* 2017;7(15).

3. Zhu W, Hou F, Fang J, et al. The role of Piezo1 in conventional aqueous humor outflow dynamics. *iScience.* 2021;24(2):102042.

4. Zhang F, Liu Y, Tang F, et al. Electrophysiological and pharmacological characterization of a novel and potent neuronal Kv7 channel opener SCR2682 for antiepilepsy. *Faseb j.* 2019;33(8):9154-9166.
